# Supplementary material for: Understanding the perceptions of parents and preschool principals on the determinants of weight management among Iranian preschoolers: A directed qualitative content analysis
Source: PLoS One. 2022 Jun 23;17(6):e0270244. doi: 10.1371/journal.pone.0270244 (PMC9223302; doi:10.1371/journal.pone.0270244)
Supplement: Attachment — Submitted filename: Response to Reviewers.doc [file pone.0270244.s002.doc]

**Response to Reviewers**

We would like to thank our dear reviewers for their time and their valuable comments. We have seriously considered and addressed all reviewer comments giving point-to-point responses. All amendments have been highlighted in yellow in the text.

**Comment:**
*1. There is inconsistent use of decimal places throughout the manuscript, i.e. in some places they are reported to two decimal places and one in others. It would be better if the authors picked one format and used it consistently.*

**Response:** Yes, of course. The format was changed **on page 3, line 55, and page 4 lines 77-8, page 7 line 146, and page 10 lines 183** as follows:

The prevalence of overweight including obesity among preschoolers was found to be 32.0% and 10.0% to 20.6% in the United States and in Europe, respectively.

In a study conducted in Tehran, the capital of Iran, it was found that the prevalence of overweight and obesity among Tehranian children aged 3-6 years was 10.3% and 4.5% in girls and 9.8% and 4.7% in boys.

| **Variables** | **Number** | **Percent** |
| --- | --- | --- |
| **Sex of participants** |  |  |
| Female | 15 | 88.3 |
| Male | 2 | 11.7 |
| **Sex of child** |  |  |
| Female | 11 | 64.7 |
| Male | 6 | 35.3 |
| **Educational status of Participants** |  |  |
| Bachelor and lower | 13 | 76.5 |
| Postgraduate education | 4 | 23.5 |
| **Occupational status** |  |  |
| Housewife | 4 | 23.5 |
| Employed | 13 | 76.5 |

Parents’ mean age was 35.1 (range: 28-42) years and principals’ mean age was 52.5 (range: 46-59) years.

**Comment:** *2. The manuscript is too long. I have made some suggestions below as to where it could be shortened.*

Agreed. The results and discussion was revised to be shorter.

**Comment:**
*3. There are numerous grammatical errors in the manuscript. I have highlighted the major issues below, but the whole manuscript would benefit from English language revision.*

Agreed. The English language was revised by an editor.

**Comment:**
*Abstract
1. It would be helpful if the authors could briefly summarize the PROCEED-PRECEDE model in the background. I understand this is challenging because of word limits but given that it is central to the analysis of the data, it seems important that readers (who may only read the abstract) understand what it is.*

**Response:** Yes, of course. The model was clarified **on page 2, lines 28-33** as follows:

The aim of the current study was to understand perceptions and experiences of Iranian parents and principals of preschool children on weight management based on the PRECEDE-PROCEED Model (PPM), a comprehensive structure for assessing health needs for designing, implementing, and evaluating health promotion and other public health programs. PRECEDE provides a structure for planning a targeted and focused public health program and PROCEED provides a structure for implementing and evaluating the program.

**Comment:**
*Background
1. Second paragraph, first sentence – there would be a better flow if the authors followed the phrase “physical and psychological disorders” in their listing of the specific disorders themselves by first listing physical disorders, then listing psychological disorders. Mixing them together, as the authors have done, is disruptive.*

**Response:** Agreed and revised **on page 3, lines 60-2** as follows:

The increased risk of physical disorders, such as cardiovascular diseases, cancer, and liver steatosis and also psychological disorders, like low self-esteem, body image concerns, depression, and weak socialization can be linked to overweight during childhood.

**Comment:** *2. Fourth paragraph, first sentence - needs grammatical revision for clarity.*

**Response:** The correction was made **on page 4, lines 74-5** as follows:

With an 83 million population, Iran that is located in Western Asia is the 18th world’s [most populous country](safari-reader://en.wikipedia.org/wiki/List_of_countries_and_dependencies_by_population).

**Comment:** *3. Fourth paragraph, last sentence – reference 18 refers to Mexican American mothers, not Iranian parents. Please revise this sentence so that this is clear.*

**Response:** The correction was made **on page 4, lines 82-4** as follows:

Therefore, understanding parents’ perceptions of their children’s weight status and its determinants are important to develop strategies and programs for weight management.

**Comment:**
*4. Sixth paragraph – the initialism PPM should be defined in full at its first mention.*

**Response:** The correction was made **on page 4, line 91** as follows:

The PRECEDE-PROCEED Model (PPM) commonly provides practical guidance for the fields of health education and health promotion.

**Comment:**
*5. Sixth paragraph – it is not clear why the authors chose to focus on the second and third phases of the PPM only. It would be helpful if they could provide some explanation for this decision – maybe this detail is more suited to the Methods section?*

**Response:** Based on the reviewer’s comment, a clarification was added **on page 4 and 5, lines 92-6** as follows:

The current study mainly focused on the second (epidemiological assessment) and third (educational and ecological assessment) phases of the PPM, to better understand the health problem and potential modifiable strategies in the Iranian family and preschool context. Other stages are also important; however, are beyond the scope of the current study due to time and tools restrictions.

**Comment:** *Methods – Study setting and participants
1. Can the authors please clarify what is meant by “The preschools were private, however, they were supervised by Tehran welfare organization”?*

**Response:** The clarification was made **on page 6, lines 136-8** as follows:

Although the preschools were supervised by Welfare Organization of Tehran, the costs of equipment and food preparation were provided by parents and preschool principals.

**Comment:**
*2. Table 1: The use of two decimal places here is unnecessary given the small numbers of participants in each of the groups. Please round up to one decimal place.*

**Response:** Agreed. The corrections were made **on page 7 line 146** as follows:

| **Variables** | **Number** | **Percent** |
| --- | --- | --- |
| **Sex of participants** |  |  |
| Female | 15 | 88.3 |
| Male | 2 | 11.7 |
| **Sex of child** |  |  |
| Female | 11 | 64.7 |
| Male | 6 | 35.3 |
| **Educational status of Participants** |  |  |
| Bachelor and lower | 13 | 76.5 |
| Postgraduate education | 4 | 23.5 |
| **Occupational status** |  |  |
| Housewife | 4 | 23.5 |
| Employed | 13 | 76.5 |

**Comment:**

*3. Table 1: It is unclear what the letters “A.s”, “B.s”, and “M.s” under the “Educational status of Participants” represent. Furthermore, is it necessary to break the participants’ educational status down to this level of detail for such a small number overall? These comments also apply to Table 2.*
**Response:** The corrections were made **on page 7 line 146 and 148** as follows:

| **Variables** | **Number** | **Percent** |
| --- | --- | --- |
| **Sex of participants** |  |  |
| Female | 15 | 88.3 |
| Male | 2 | 11.7 |
| **Sex of child** |  |  |
| Female | 11 | 64.7 |
| Male | 6 | 35.3 |
| **Educational status of Participants** |  |  |
| Bachelor and lower | 13 | 76.5 |
| Postgraduate education | 4 | 23.5 |
| **Occupational status** |  |  |
| Housewife | 4 | 23.5 |
| Employed | 13 | 76.5 |

| **Participants** | **Age** | **Sex** | **Educational level** | **Work experience** |
| --- | --- | --- | --- | --- |
| **Principal 1** | 59 | female | Bachelor | 35 |
| **Principal 2** | 46 | female | Bachelor | 15 |

**Comment:**
*Methods – Data collection
1. Sentence three is very long and difficult to follow.*

**Response:** Agreed. The sentence was omitted and moved to Table 3. **Page 8, lines 164.**

**Comment:**
*2. It is not necessary to provide the specific training and experience of the interviewer, suffice to say “trained researcher”.*

**Response:** The correction was made **on page 8, lines 156-7** as follows:

The interviews were executed by the trained researcher in the preschool setting and lasted 15 to 35 minutes.

**Comment:**
*3. Table 3: Question 1 (“How is your children’s eating status?”) appears to have translated poorly into English. Could the authors please clarify is this is a correct translation?*

**Response:** The clarification was made **on page 8, line 164** as follows:

1. Please explain your child’s nutrition status.

**Comment:**
*Methods – Data analysis
1. First sentence – repetition regarding participants consent for audio recording, please delete.*

**Response:** Based on the reviewer’s comment, **on page 8, line 167-8 w**as revised as follows:

Audio recording of the interviews was done by the first author and then, they were transcribed verbatim.

**Comment:**
*2. There is no mention of the phrase “direct content analysis” in this section, as is mentioned in the Abstract. This should be included so that the reader can be clear on the type of analysis used by the researchers.*

**Response:** Agreed. The correction was made **on page 9, lines 170-1** as follows:

In line with the goals of the study, the researchers utilized the approach developed by Hsieh and Shannon [22] for directed content analysis.

**Comment:**
*Results
1. First paragraph, last sentence is an overstatement. The authors cannot factually state that the data were saturated, only that they considered it to be so. Please revise.*

**Response:** The suggested correction was made **on page 10 and 11, lines 190-4** as follows:

Data saturation was considered when the last couple of interviewees did not add new perceptions and sufficient data had been obtained regarding the object [23]. After 16 interviews, the codes were repeated and no new codes were found and the data were considered to be saturated and data collection was stopped.

**Comment:**
*2. The results section is very long. Firstly, there is repetition regarding the PPM model itself littered throughout the entire results section. The manuscript could be shortened considerably if this were removed. Secondly, it may be beneficial for the authors to consider referring to Table 4 rather than repeating quotes in the table and text.*

**Response:** As suggested, the results section was shortened and repetitions were omitted.

**Comment:** *3. Section 4.1.1.2 – the points here regarding apartment living do not appear to fit with this code (Parents’ and principals’ attitude). They were already mentioned at section 2.2.4 but would also seem relevant under section 3.1.1. as they are related to the environment.*

**Response:** Thank you for your comment. We agree that apartment living do not fit with Parents’ and principals’ attitude, however we believe it is related to “Action of neighbors”.

**Comment:**
*4. Section 4.2 (Reinforcing factors) – please rephrase “According to the participants…” at sentence one here. It was the researchers, not the participants, who were responsible for the analysis.*

**Response:** Agreed and revised **on page 18 lines 361-3 and page 20, lines 400-3** as follows:

**4.2. Enabling factors**

According to the data analysis, "parents’, principals’, and teachers’ skills, rules and laws in the preschools, and also availability" were placed under the enabling factors.

**4.3. Reinforcing factors**

According to the data analysis, “family support and effects, teachers’ encouragement and influences, and peers’ influences” were under the subcategory of feedback, encouragement, and influence of others and as reinforcing factors of weight management.

**Comment:** *Discussion
1. This entire section needs substantial revision. There is frequent repetition of the results in the discussion, with little integrated discussion of findings of a whole. Rather than listing every finding in isolation, it would be helpful if the authors focused on what they consider to be the key results and how they may relate to each other.*
**Response:** The discussion was revised accordingly **on page 22-24 lines 437-480.**

**Discussion**

The aim of this study was to explore the perception and experiences of children’s parents and principals regarding genetic, behavioral, environmental, and also predisposing, reinforcing and enabling factors on weight management in Iranian preschoolers. Based on our knowledge, these factors have not yet been explored from the point of view of Iranian children’s parents and principals.

The first aim of the study was to investigate these factors from the point of view of parents. This study identified genetic, behavioral (e.g., food preferences, not eating some foods due to allergy or stomach reflux and also physical activity and sedentary behaviors as children’s actions) and (e.g., the effect of parents’, peers’, principals’ and teachers’ behavior and also influence of grandparents’ and neighbors’ behaviors as actions of others) and environmental (e.g., home, grandparents’ home and preschool) factors from the epidemiological construct. Also, predisposing (e.g., child’s attitude, parent’s and principals’ attitude, as well as parents’ knowledge and parents’ and principals’ beliefs), enabling (e.g., parental skills and skills of the principals and teachers, rules and laws in the preschools, and availability), and reinforcing (e.g., family support and influences, teachers’ encouragement and influences, and peers’ influences) factors were identified from the educational and ecological construct. Some of these factors (e.g., genetic, food preferences, physical activity, sedentary behaviors, the effect of the behavior of parents, peers, teachers, and grandparents, parent’s attitude and knowledge) have also been revealed in previous studies in children [24-35]. These similarities maybe indicate that these factors have a crucial effect on weight management promotion among preschoolers. Differences between some of our findings and other studies could be due to different cultures of families and the preschool’s environment.

The second aim of the study was to investigate the factors from the point of view of preschool principals. Most of the identified factors by parents were also reported by principals; however, principals also stated factors that were not reported by the parents. Additional factors reported by principals were caring about children’s health and diet in the preschool, existing rules in preschools, and the influence of the weather on children’s play. The combination of perspectives of parents and principals can be used to design an educational intervention for weight management promotion among preschoolers.

In general, according to our findings, parents, especially mothers, at home environment and principals at preschool play a crucial role to promote preschoolers’ weight management. Consequently, future interventions for preschoolers’ weight management promotion should contain multiple components: one component focusing on mothers at home and one component focusing on principals at preschool. This is in line with studies reporting that multiple-component interventions are more effective than one-component interventions [36, 37]. For instance, the home component can be considered to increase mothers’ skills and knowledge and be a good role model for children, whereas the preschool component can focus on the promotion of principals’ skills and teachers’ role models.

In the current study, some mothers reported that their mood and also the parents-children relationship quality can affect their children’s eating and physical activity behaviors. A body of evidence supports this finding; there is an association between the quality of the parent-child relationship and obesogenic behaviors. Also, the emotional space created by parents may affect the association between parental practices and weight-related behaviors [38, 39].

**Comment:** *Conclusion
1. The conclusion section should report the major takeaway findings of the study, not it’s novelty. It would be helpful if the authors outlined what specifically they consider to be the important sociocultural information provided by the study?*

**Response:** Agreed and revised **on page 24, lines 488-494 and also page 2 and 3 lines 46-51** as follows:

**Conclusion**

Parents’ and principals’ experiences regarding preschoolers’ weight management promotion confirm the genetic, behavioral, environmental, predisposing, enabling, and reinforcing factors of the PPM. Additionally, in the current study, “quality of child-parent relationship” was determined as a new factor affecting preschoolers’ weight management promotion; however, it was not in the PPM. This finding may be related to culture and family relationship type among Iranian people and is suggested to be investigated in future studies.

**Comment:**

*Reviewer #2: Authors have done a good job, but there are few things which need to be modified as suggested below. The authors should explain their choice of qualitative data collection, e.g why in-depth interviews and not focus group discussion and how they ended up with 15 participants. Its understandable that in qualitative we don't have sample size but at least it can be explained how the ended with only 15 as targeted (was there a saturation point?). I found the strengths and limitations sections difficult to understand, and the conclusion. These need to be thought through and improved*

**Response:** Thanks for your comment.

Firstly, in qualitative studies data collection method is not predetermined and fixed and is influenced by such factors as objects of the study, participant’s willingness and their cultural background. In the current study, participants were inclined to individual interviews. In addition, Iranian mothers culturally tend to be reluctant to discuss their children's behavior in public. Secondly, depth interviews were chosen above other methods to get enough detail.

**Response:** The suggested correction was made **on page 10 and 11, lines 190-4** as follows:

Data saturation was considered when the last couple of interviewees did not add new perceptions and sufficient data had been obtained regarding the object [23]. After 16 interviews, the codes were repeated and no new codes were found and the data were considered to be saturated and data collection was stopped.

**Response:** Agreed and revised **on page 264 lines 481-494 and also page 2 and 3 lines 46-51** as follows:

**Strengths and limitations**

The main strength of the present study is the fact that preschool principals were interviewed in addition to parents. Additionally, this study identified participants’ attitude, believes, and behaviors that could be targeted in designing future interventions. One of the limitations of this study is that only two fathers were interviewed. Although in Iran, mothers play an important role in shaping the behaviors of their children, fathers also affect their behaviors in the home environment.

**Conclusion**

Parents’ and principals’ experiences regarding preschoolers’ weight management promotion confirm the genetic, behavioral, environmental, predisposing, enabling, and reinforcing factors of the PPM. Additionally, in the current study, “quality of child-parent relationship” was determined as a new factor affecting preschoolers’ weight management promotion; however, it was not in the PPM. This finding may be related to culture and family relationship type among Iranian people and is suggested to be investigated in future studies.
