# Supplementary material for: Understanding the perceptions of parents and preschool principals on the determinants of weight management among Iranian preschoolers: A directed qualitative content analysis
Source: PLoS One. 2022 Jun 23;17(6):e0270244. doi: 10.1371/journal.pone.0270244 (PMC9223302; doi:10.1371/journal.pone.0270244)
Supplement: Attachment — Submitted filename: PONE.docx [file pone.0270244.s003.docx]

PONE-D-20-18528R1

Summary

The paper focuses on addressing obesity among preschoolers in Iran by engaging parents and principals to identify modifiable risk factors using the PRECEDE/PROCEED Planning model. The background literature points to the need to examine this issue as there are limited studies focused on improving preschool children’s health and risk for obesity. Overall, the paper provides important insights on this topic and I like the approach taken by using the PPM.

Major issue

1. A major issue is that the paper still includes many grammatical errors and needs to be revised by an editor to improve clarity
2. Also, I am unclear about the use of the same questions for parents/principals- wouldn’t the questions need to different for principals as educators not parents?

Minor issues

Abstract

1. Results- reword “Obese children” to children with obesity.
2. Results may be better understood if described within the PRECEDE sections.

Background

1. Lines 106, 109 – Sentences needs citations

Study design

1. Need to cite content analysis approach (line 125)
2. Line 127 We used individual semi-structured interviews with open-ended questions, and assessed play equipment and documents, such as the diet and physical activity plans in preschools. This triangulation method increases the credibility and conformability of the study and the understanding of various aspects of weight management promotion
3. Line 136 Preschools in six areas of Tehran
4. line 137 supervised by the Welf
5. Line 142 remove the following it is unclear “and if needed, the fathers were also to continue interview.”
6. Line 143 add being a principal as criterion
7. Line 153 change to Interview questions
8. Line 158 After the participants’ answered, the researcherutilized probe questions to explore participants’ experiences of the concepts by asking some questions, such as “What do you mean?, Please explain more about …?, Would you give me an example to understand what you mean?”. After transcription and review of some interviews, to clarify ambiguities, the researcher called the participants and asked them for more details.
9. Line 164 Table 3. Interview Questions – how did you ask the principals the questions were they revised to fit their job vs. their child?
10. Be consistent with wording- first author or main researcher?

Results

1. This section could benefit from a figure to show findings based on PPM
2. Line 221 if weather was good
3. Line 237 Parents also reported ~~that was~~ they were responsible to take their children to the preschool by car or on foot.
4. Line 426 Quality of child-parent~~s~~ relationship
