# Supplementary material for: Understanding the perceptions of parents and preschool principals on the determinants of weight management among Iranian preschoolers: A directed qualitative content analysis
Source: PLoS One. 2022 Jun 23;17(6):e0270244. doi: 10.1371/journal.pone.0270244 (PMC9223302; doi:10.1371/journal.pone.0270244)
Supplement: Attachment — Submitted filename: Response to the Reviewer .doc [file pone.0270244.s004.doc]

**Response to the Reviewer**

We would like to thank the reviewer for her/him time and valuable comments. We have seriously considered and addressed all the reviewer’s comments giving point-to-point responses. All amendments have been highlighted in yellow in the text.

**Comment:**
**Major issue**

1. A major issue is that the paper still includes many grammatical errors and needs to be revised by an editor to improve clarity.

**Response:** Agreed. The English language was revised by an editor.

2. Also, I am unclear about the use of the same questions for parents/principals- wouldn’t the questions need to different for principals as educators not parents?

**Response:** Indeed questions differed between the groups of respondents. This was clarified by adding an audience column **on page 8, line 166** as follows:

Table 3: Interview Questions

| **Constructs of PPM** | **Interview Questions** | **Audiences** |
| --- | --- | --- |
| **Behavioral factors** | 1. Please explain your child’s nutrition status. | parents |
|  | 2. Please explain your child/ children’s physical activity on weekdays/ weekends. | parents/principals |
|  | 3. How do you manage your child/ children’s weight status? | parents/principals |
| **Predisposing factors** | 4. How do you assess your children’s weight? (Based on the practitioner’s assessment or your own assessment) | parents |
|  | 5. Why do you manage your child/ children’s weight status? | parents/principals |
|  | 6. What is your belief regarding the weight status of children aged 3-5 years? | parents/principals |
| **Enabling factors** | 7. Please tell me about the rules in your home to manage your child’s weight. | parents |
|  | 8. Please tell me about diet and physical activity plans in the preschool. | parents/principals |
|  | 9. What are the rules in the preschool to improve children’s weight management? | principals |
|  | 10. What problems do you encounter to manage your child’s weight? | parents |
| **Reinforcement factors** | 11. Please tell me how you influence the dietary intake and physical activity of your children. | parents |
|  | 12. Please tell me how teachers and peers influence your child/ children’s eating habits and physical activity. | parents/principals |

**Minor issues**

**Comment:**

**Abstract**

1. Results- reword “Obese children” to children with obesity.

**Response:** Agreed. Based on your next comment, the sentences were revised completely.

2. Results may be better understood if described within the PRECEDE sections.

**Response:** Agreed and revised **on page 2 lines 36-46** as follows:

This study identified genetic, behavioral (e.g., food preferences, physical activity, sedentary behaviors, the effect of parents’, peers’, principals’ and teachers’ behavior and influence of grandparents’ and neighbors’ behaviors) and environmental (e.g., home, grandparents’ home and preschool) factors from the epidemiological construct. Additionally, predisposing (e.g., child’s attitude, parent’s and principals’ attitude, as well as parents’ knowledge and parents’ and principals’ beliefs), enabling (e.g., parental skills and skills of the principals and teachers, rules and laws in the preschools, and availability), and reinforcing (e.g., family support and influences, teachers’ encouragement and influences, and peers’ influences) factors were identified from the educational and ecological construct.

**Background**

1. Lines 106, 109 – Sentences needs citations

**Response:** Of course. The citations were added **on page 5, lines 106-109**.

**Study design**

1. Need to cite content analysis approach (line 125)

**Response:** Of course. The citations was added **on page 6, lines 125**.

2. Line 127 We used individual semi-structured interviews with open-ended questions, and assessed play equipment and documents, such as the diet and physical activity plans in preschools. This triangulation method increases the credibility and conformability of the study and the understanding of various aspects of weight management promotion

**Response:** The correction was made **on page 6, lines 126-130** as follows:

We used individual semi-structured interviews with open-ended questions, and assessed play equipment and documents, such as the diet and physical activity plans in preschools. This triangulation method increases the credibility and conformability of the study and the understanding of various aspects of weight management promotion.

3. Line 136 Preschools in six areas of Tehran
4. line 137 supervised by the Welf
5. Line 142 remove the following it is unclear “and if needed, the fathers were also to continue interview.”
6. Line 143 add being a principal as criterion
7. Line 153 change to Interview questions

**Response:** Agreed. All of mentioned errors were corrected **on pages 6, 7 and 8, lines 135, 137, 141, 142, and 155**.

8. Line 158 After the participants’ answered, the researcher utilized probe questions to explore participants’ experiences of the concepts by asking questions, such as “What do you mean?, Please explain more about …?, Would you give me an example to understand what you mean?”. After transcription and review of some interviews, to clarify ambiguities, the researcher called the participants and asked them for more details.

**Response:** Agreed and revised **on page 8, lines 159-164** as follows:

After the participants answered, the researcher utilized probe questions to explore participants’ experiences of the concepts by asking some questions, such as “What do you mean?, Please explain more about …?, Could you give me an example to understand what you mean?”. After transcription and review of some interviews, to clarify ambiguities, the researcher called the participants and asked them for more details.

9. Line 164 Table 3. Interview Questions – how did you ask the principals the questions were they revised to fit their job vs. their child?

**Response:** We didn’t ask the principals regarding fitting principals’ job vs. their child since there is not any questions to fit principals’ job vs. their child in PRECEDE-PROCEED model. We just asked them “Please tell me how teachers and peers influence your child/ children’s eating habits and physical activity”.

10. Be consistent with wording- first author or main researcher?

**Response:** Agreed. First author and main researcher were replaced by the researcher in the manuscript **on page 6, 8 and 9, lines 120, 160,163, 168, and 172**.

**Results**

1. This section could benefit from a figure to show findings based on PPM

**Response:** as suggested a figure was designed and added **on page 22, line 439** as follow:

**Figure 1: PRECEDE-PROCEED model to understand perceptions and experiences of Iranian parents and principals of preschool children on weight management**

2. Line 221 if weather was good
3. Line 237 Parents also reported ~~that was~~ they were responsible to take their children to the preschool by car or on foot.
4. Line 426 Quality of child-parent~~s~~ relationship

**Response:** Agreed. All of mentioned comments were corrected **on pages 12 and 13, 21 lines 223, 239, and 429.**
