# Supplementary material for: Understanding the perceptions of parents and preschool principals on the determinants of weight management among Iranian preschoolers: A directed qualitative content analysis
Source: PLoS One. 2022 Jun 23;17(6):e0270244. doi: 10.1371/journal.pone.0270244 (PMC9223302; doi:10.1371/journal.pone.0270244)
Supplement: Attachment — Submitted filename: Comments.docx [file pone.0270244.s005.docx]

I would like to congratulate the author. The author has addressed all the comments well. I don’t have any further comments
