# Supplementary material for: Understanding the perceptions of parents and preschool principals on the determinants of weight management among Iranian preschoolers: A directed qualitative content analysis
Source: PLoS One. 2022 Jun 23;17(6):e0270244. doi: 10.1371/journal.pone.0270244 (PMC9223302; doi:10.1371/journal.pone.0270244)
Supplement: Attachment — Submitted filename: Response to the Reviewer.doc [file pone.0270244.s006.doc]

**Response to the Reviewer**

We would like to thank the editor for his/her time and valuable comments. We have seriously considered and addressed all the editor’s comments giving point-to-point responses. All amendments have been indicated by track changes in the text.

**Introduction**

**Comment:**
"With a population of 83 million people, Iran, that is located in Western Asia, is the 18th world’s 18th most populous country.”

**Response:** The correction was made **on page 4, lines 74-75** accordingly:

With a population of 83 million people, Iran, that is located in Western Asia, is the world’s [18th most populous country](safari-reader://en.wikipedia.org/wiki/List_of_countries_and_dependencies_by_population).

**Study setting and participants**

**Comment:**

“…The inclusion criterion was being a principal or parent of preschoolers aged 3-5 years.”

(Note: This change does not apply if the criterion did indeed specify that they had to be both a principal and a parent, although I suspect this is not the case.)

**Response:** Agreed. The correction was made **on page 7, lines 142** accordingly:

The inclusion criterion was being a principal or parent of preschoolers aged 3-5 years.

**Results**

**3.1.3. Preschool**

**Comment:**

Children spend most of the days in a week and most of the hours in a day in the preschool and were eating breakfast, lunch, and snacks there.

**Response:**  The correction was made **on page 16, lines 309** accordingly:

Children spend most of the days in a week and most of the hours in a day in the preschool and were eating breakfast, lunch, and snacks there.

**4.1.1.2. Parents’ and principals’ attitude**

**Comment:**

Some mothers tended that their children were obese, and they were not dissatisfied with their children’s weight gain and eating behaviors. "She is underweight and weak; she is slim …"

(Note: Please use a different phrase here as "tended" does not make any sense in this context.)

**Response:** Agreed. The correction was made **on page 18, lines 346** as follow:

Some mothers were inclined to have obese children, and they were not dissatisfied with their children’s weight gain and eating behaviors.
